# Genome-wide association study of response to tumour necrosis factor inhibitor therapy in rheumatoid arthritis

**DOI:** 10.1038/s41397-018-0040-6

**Published:** 2018-08-31

**Authors:** Jonathan Massey, Darren Plant, Kimme Hyrich, Ann W. Morgan, Anthony G. Wilson, Athina Spiliopoulou, Marco Colombo, Paul McKeigue, John Isaacs, Heather Cordell, Costantino Pitzalis, Anne Barton, BRAGGSS, MATURA Consortium

**Affiliations:** 1 Arthritis Research UK Centre for Genetics and Genomics, Centre for Musculoskeletal Research, The University of Manchester, Manchester, UK; 2 NIHR Manchester Biomedical Research Centre, Manchester University NHS Foundation Trust, Manchester Academic Health Science Centre, Manchester, UK; 3 Arthritis Research UK Centre for Epidemiology, Centre for Musculoskeletal Research, The University of Manchester, Manchester, UK; 4 School of Medicine, University of Leeds, and NIHR Leeds Biomedical Research Centre, Leeds Teaching Hospitals NHS Trust, Leeds, UK; 5 School of Medicine & Medical Science, Conway Institute, University College Dublin, Belfield, Dublin, Ireland; 6 Centre for Population Health Sciences, Usher Institute, University of Edinburgh, Edinburgh, UK; 7 Pharmatics Ltd, 9 Little France Rd, Edinburgh, UK; 8 Institute of Cellular Medicine, Newcastle University, and NIHR Newcastle Biomedical Research Centre, Newcastle upon Tyne Hospitals NHS Foundation Trust, Newcastle upon Tyne, UK; 9 Institute of Genetic Medicine, Newcastle University, Newcastle upon Tyne, UK; 10 William Harvey Research Institute, Barts and The London School of Medicine & Dentistry and Barts Health NHS, Queen Mary University of London, London, UK

## Abstract

Rheumatoid arthritis (RA) is characterised by chronic synovial joint inflammation. Treatment has been revolutionised by tumour necrosis factor alpha inhibitors (TNFi) but each available drug shows a significant non-response rate. We conducted a genome-wide association study of 1752 UK RA TNFi-treated patients to identify predictors of change in the Disease Activity Score 28 (DAS28) and subcomponents over 3–6 months. The rs7195994 variant at the *FTO* gene locus was associated with infliximab response when looking at a change in the swollen joint count (SJC28) subcomponent (*p* = 9.74 × 10^−9^). Capture Hi-C data show chromatin interactions in GM12878 cells between rs2540767, in high linkage disequilibrium with rs7195994 (*R*^2^ = 0.9) and *IRX3*, a neighbouring gene of *FTO*. *IRX3* encodes a transcription factor involved in adipocyte remodelling and is regarded as the obesity gene at the *FTO* locus. Importantly, the rs7195994 association remained significantly associated following adjustment for BMI. In addition, using capture Hi-C data we showed interactions between TNFi-response associated variants and 16 RA susceptibility variants.

## Introduction

Rheumatoid arthritis (RA) is a chronic autoimmune disorder affecting around 1% of the United Kingdom (UK) population [1], which leads to synovial joint inflammation and joint damage. Tumour necrosis factor (TNF) is a pro-inflammatory cytokine essential for immunity to infections; however, its dysregulation is important in the pathogenesis of RA and many other chronic inflammatory diseases [2]. It is primarily, but not exclusively, produced by activated macrophages and exerts its function through binding to its cognate receptors: TNFR1 and TNFR2 [3]. There are a number of TNF inhibitor (TNFi) biologics and biosimilars licensed for treatment of RA within the UK: Infliximab (Remicade, biosimilars: Remsima and Inflectra), adalimumab (Humira), certolizumab pegol (Cimzia), and golimumab (Simponi) are antibodies or fragments of antibodies that are targeted towards the TNF, whilst etanercept (Enbrel, biosimilar: Benapali) comprises two TNFR2 extra-cellular domains fused to the IgG1 Fc.

Response to TNFi treatment varies between individuals, with as many as 30–40% showing no clinical improvement [4]. Given the expense of these drugs (around 5000–10,000 GBP per patient per year in the UK) and the potential for detriment to non-responding patients, the identification of predictors of response from pre-treatment (baseline) characteristics would be of great clinical, societal and economic benefit. The importance of this question motivated the formation of the UK MAximizing Therapeutic Utility in RA (MATURA) consortium [5], which has the wider remit of using blood-based biomarkers and synovial pathobiology to inform the stratification of all stages of RA treatment.

Genetic variants are stable characteristics that can be easily measured on a genome-wide scale. Previous genome-wide association studies (GWASs) of response to TNFi agents in RA have identified the *CD84* [6] and *PDE3A-SLCO1C1* [7] loci as the strongest candidates but reproducibility has been an issue [8] and recent studies have questioned the utility of studying genetic variation in relation to response to TNFi in RA [9]. However, previous studies have been limited by small sample sizes, different population sub-groups, and inconsistent measures of response and follow-up times.

In the current study, we present a large GWAS of TNFi response in UK RA patient samples, which were all collected using the same study protocol and analysed using not only the composite measure of response (change in disease activity score in 28 joints (DAS28)) but also the subcomponent of the score.

## Methods

### Patients

The British Society for Rheumatology Biologics Register for Rheumatoid Arthritis (BSRBR-RA) was established with the aim of assessing the safety of biologic agents in the treatment of RA and has collected detailed clinical and response data (www.bsrbr.org). Collaborations have been established between some of the larger referring centres to form the Biologics in Rheumatoid Arthritis Genetics and Genomics Study Syndicate (BRAGGSS; www.braggss.co.uk), which collects blood samples from biologic treated RA patients for DNA, RNA and cellular studies. Contributing patients provide written informed consent, and the BRAGGSS study is ethically approved (COREC 04/Q1403/37).

For the current study, clinical measurements collected included swollen 28-joint count (SJC28), tender 28-joint count (TJC28), erythrocyte sedimentation rate (ESR) and patient global health assessment (PGA), as measured by visual analogue scale (VAS). Measurements of these were taken at baseline and again at follow-up (between 3 and 6 months).

Patients were selected for genotyping if they were currently receiving or about to begin receiving treatment with a TNFi. Patients were excluded if they stopped treatment within the first 6 months for reasons other than lack of efficacy. Of the 1770 patients available for this study: 76.8% were female, 92.8% were receiving their first biologic treatment, and other baseline characteristics are shown in Table 1.

**Table 1:** Baseline patient characteristics

Baseline measure	Mean (standard deviation)	Range
Age	56.9 years (11.36)	19.6–85.3
Disease duration	12.4 years (9.92)	0.09–54.8
DAS28 score	6.35 (0.97)	1.72–9.2
SJC28	10.59 (6.12)	0–28
TJC28	15.94 (7.27)	0–28
ESR	37.19 mm/h (28.16)	0.18–137
PGA	71.7 VAS measure (18.87)	0–100

Four component disease activity scores (DAS28-ESR) (0.56(√TJC28) + 0.28(√SJC28) + 0.70(ln(ESR)) + 0.014 × PGA) were calculated for each individual at baseline and follow-up. Where this was not possible, due to missing PGA in a small number of cases, a three component DAS28 was calculated as follows: [0.56(√TJC28) + 0.28(√SJC28) + 0.70(ln(ESR))] × 1.08 + 0.16.

### Genotyping

Genotype data from 1770 samples was generated from several Affymetrix and Illumina arrays: Affymetrix GeneChip Mapping 500K Array Set (presented previously [10]), Affymetrix Genome-Wide Human SNP Array 6.0, Illumina HumanHap550/Illumina HumanHap650, Illumina OmniExpress, and Illumina HumanCoreExome (Supplementary Table 1). Standard QC was conducted on each individual array: SNPs and samples were excluded if there was >2% missing data, and SNPs with MAF < 0.01 and Hardy Weinberg Equilibrium (HWE) *p* < 1 × 10^−4^ were also excluded.

Ideally for imputation, the intersection of SNPs from across all arrays would be used but this yielded < 10,000 SNPs. Therefore, the arrays were grouped as follows, showing the number of SNPs available for imputation: Affymetrix group (272 006 SNPs), IlluminaHumanHap group (460 812 SNPs) and the Illumina Omni-HumanCore group (204 894 SNPs).

The three groups were then imputed separately to the Haplotype Reference Consortium (release 1.1) [11] using the Sanger Imputation Service (https://imputation.sanger.ac.uk/). The pre-phasing was performed using SHAPEIT2 [12] and imputation using the PBWT algorithm [13].

Imputed files were filtered to exclude variants with an INFO score <0.8. The files were then subset to include only the variants common to all three groups (9,814,288 SNPs). GCTA [14] was used to conduct principal component analysis (PCA) and generate a genetic relationship matrix (GRM). These were used to exclude samples of non-Caucasian ethnicity and samples that showed genetic relatedness (GRM cut-off 0.05).

### Statistical analyses

Genotype data from the three separate imputation groups were analysed for change in outcome from baseline to follow-up (baseline measure minus 3–6 month follow-up measure, where the baseline measure is taken before the start of TNFi treatment) of DAS28 (calculated using ESR), SJC28, TJC28, ESR and PGA. These will be termed ΔDAS28-ESR, ΔSJC28, ΔTJC28, ΔESR and ΔPGA, respectively (the raw distributions of these measures are shown in Supplementary Figure 1).

First, a combined analysis including all TNFi was performed, followed by the three largest drug groups independently: adalimumab (Humira), etanercept (Enbrel), and infliximab (Remicade).

SNPTEST v2.5.2 [15] was used to implement the linear regression of these measures assuming an additive genetic model. All measures were mean-centred and scaled to have a variance of 1 within each group before the linear regression. The following covariates were used in the linear regression: first three principal components, the baseline measure (DAS28-ESR, SJC28, TJC28, ESR or PGA at commencement of treatment—also mean-centred and scaled to have a variance of 1), sex, concurrent disease-modifying anti-rheumatic drugs (DMARDs), and imputation group (described above). Imputed genotype uncertainty was dealt with using a missing data likelihood score test (“−score” option). Only SNPs with a minor allele frequency (MAF) ≥ 0.05 in each group were considered for the primary analysis. Low-frequency SNPs (MAF ≥ 0.01 < 0.05) were considered in a secondary analysis. SNP associations at the <5 × 10^−5^ *p*-value threshold were considered suggestive of association.

### Chromosome conformation capture data

Chromosome conformation capture technology (Capture Hi-C) data from various sources [16–18] were used to investigate chromatin interactions between associated variants and distant chromosomal regions. This technology can help in the selection of candidate genes and provide mechanistic insights at associated loci, as has been demonstrated previously [17, 19]. Capture HiC Plotter (CHiCP) was used for data visualisation [20].

### Heritability estimation

We estimated the heritability of response to TNFi, as measured by the change in DAS28 components between baseline and 6-month follow-up: Δlog(ESR), Δ√SJC28, Δ√TJC28, ΔPGA. To make each response measure as homogeneous as possible, we excluded samples that were only followed-up at 3 months. Each outcome was adjusted for: first ten principal components, baseline measure, sex, concurrent DMARDs, genotyping array, and study. Heritability was estimated using the rank-transformed residuals from linear regression of each outcome on the covariates.

We estimated heritability using GCTA [14] and a Bayesian hierarchical model. The asymptotic large-sample approximations underlying the REML estimation used in GCTA are unlikely to hold with the modest sample size available for this study [21]. For this reason, in addition to GCTA, we used a fully Bayesian method that does not depend on large-sample approximations and returns the posterior distribution of heritability. The Bayesian model was implemented using JAGS (http://mcmc-jags.sourceforge.net) and its R interface package rjags. Inference was performed using Markov chain Monte Carlo sampling (see Supplementary Information 1 for details).

## Results

A total of 1752 samples passed quality control, with the maximum number available for analysis depending on the trait: 1723 for ΔSJC28/ΔTJC28, 1709 for ΔPGA, 1569 for ΔDAS28 (based on ESR), and 1514 for ΔESR (Table 2). There are fewer ΔESR than ΔDAS28-ESR as the calculated DAS28-ESR was sometimes available from clinical notes without the individual component values. The study had >80% power to detect a difference in the DAS28 of ≥0.6 units (a clinically meaningful change as defined by EULAR response criteria) for allele frequencies of ≥5%.

**Table 2:** Sample numbers post-QC

TNFi	Phenotype, *n* (imputation group 1 / 2 / 3)				
	ΔDAS28	ΔESR	ΔSJC28	ΔTJC28	ΔPGA
Adalimumab	500 (171/284/45)	487 (162/282/43)	551 (169/339/43)	551 (169/339/43)	549 (167/339/43)
Etanercept	564 (309/243/12)	549 (297/240/12)	619 (304/303/12)	619 (304/303/12)	615 (301/302/12)
Infliximab	441 (331/56/54)	414 (310/53/51)	434 (319/63/52)	434 (319/63/52)	426 (313/62/51)
Other	64 (0/64/0)	64 (0/64/0)	119 (0/119/0)	119 (0/119/0)	119 (0/119/0)
**Total**	**1569** **(811/647/111)**	**1514** **(769/639/106)**	**1723** **(792/824/107)**	**1723** **(792/824/107)**	**1709** **(781/822/106)**

Across all drugs and outcomes, studying variants with a MAF > 5%, five loci had *p* < 1 × 10^−5^, with one, rs7195994 mapping to the *FTO* gene locus, surpassing genome-wide significance (*p* < 5 × 10^−8^) (Table 3). Lower frequency variants are presented in Supplementary Table 2. For all of the SNPs with a MAF > 5%, there was evidence of association (*p* < 0.01) at the same SNP in at least one other outcome and/or TNFi agent (Supplementary Table 3). The Quantile–Quantile (QQ) plots for each analysis are shown in Supplementary Figure 2.

**Table 3:** Association analysis results (*p* < 1x 10^−7^) for variants with MAF ≥ 5 %

Pheno	TNFi	Gene region	SNP	Chr	BP	A1	A2	Genotype counts	MAF	P	Beta	SE
SJC28	Infliximab	intronic FTO	rs7195994	16	54060205	G	A	363/68/3	0.085	9.74E-09	−0.48	0.08
SJC28	Adalimumab	intergenic BRINP1/LINC01613	rs10739537	9	122188604	G	T	70/275/206	0.377	9.11E-08	−0.21	0.04
PGA	Etanercept	intergenic MMP20/MMP27	rs948138	11	102501665	G	A	132/292/191	0.452	7.62E-08	0.25	0.05
TJC28	Infliximab	intergenic C10orf90/DOCK1	rs11599217	10	128566964	G	T	128/210/96	0.463	7.27E-08	−0.29	0.05
TJC28	All	intronic ZNF595,ZNF718	rs2187874	4	82321	G	T	1279/397/47	0.142	7.00E-08	−0.19	0.04

### All TNFi combined

One variant, rs2187874 intronic in the *ZNF595* / *ZNF721* genes (Supplementary Figure 3a) was associated with ΔTJC28 phenotype, (*p* = 7 × 10^−8^). The variant has chromatin interactions in the GM12878 data [18] with *PIGG* and *ZNF721* genes (Supplementary Figure 3b) but neither gene is an obvious candidate and findings will require replication.

### Infliximab

rs7195994 on chromosome 16, intronic in the *FTO* gene, was associated with response to infliximab when analysing ΔSJC28 (p = 9.74 × 10^−9^) (Supplementary Figure 4a). *FTO* encodes a 2-Oxoglutarate–Dependent Nucleic Acid Demethylase and is associated with obesity [22] and Body Mass Index (BMI) [23]. rs7195994 is in LD with rs2540767 (*r*^2^ = 0.9), which shows chromatin interaction with the *IRX3* gene [18] (Supplementary Figure 4b), which some studies have suggested is one of the causal genes for obesity/BMI at the *FTO* locus [24]. However, the SNP presented here remains associated with response to infliximab at the genome-wide level even after adjusting for BMI. Furthermore, there was no association in these samples using a linear regression (including the same covariates as the GWAS) between ΔSJC28 and a weighted genetic risk score (wGRS) calculated from the effect sizes for SNPs associated with both BMI and waist circumference in two studies [25, 26].

A variant, rs11599217, showing chromosome interactions with the *DOCK1* gene was significantly associated with ΔTJC28 (*p* = 7.27 × 10^−8^) (Supplementary Figure 5). DOCK1 (also called Dock180) functions with the ELMO1 protein to regulate the actin cytoskeleton during phagocytosis (through the small GTPase Rac) [27].

### Adalimumab

The most significant association in the adalimumab treated subgroup was for the rs10739537 variant on chromosome 9 and ΔSJC28 phenotype (*p* = 9.11 × 10^−8^). The variant is located upstream of the *BRINP1* (also called *DBC1*) gene (Supplementary Figure 6). Interestingly, DBC1 is an essential subunit of the FOXP3 complex in human CD4+ regulatory T-cells (Tregs) [28] and these cells are thought to be the major driver of RA susceptibility.

### Etanercept

The only locus with *p* < 1 × 10^−7^ when analysing the ΔPGA phenotype across all drugs was in etanercept. rs948138 (*p* = 7.62 × 10^−8^) (Supplementary Figure 7a), shows chromatin interactions in a number of cell types (Macrophages, Naive B-cells, Naive CD4+, Naive CD8+ and Activated CD4+) with the promoter of *BIRC2* [16] (Supplementary Figure 7b).

### Low frequency variants

There were 14 variants with a MAF < 5% but ≥1% that were associated *p* < 1 × 10^−7^ (Supplementary Table 2). However, these were considered separately due to both limited power and difficultly, despite INFO scores >0.8, in ascertaining accuracy. To add potential support to such SNPs, we looked for evidence of association (*p* < 0.01) in other outcomes and other TNFi agents (Supplementary Table 3). The only SNP without such support was rs34619498, intronic in *EMCN*.

The SNP in *RAD51B* is of particular note given that this locus is associated with RA susceptibility. Interestingly, using in-house capture-HiC data [17] from GM12878 cells, we can show interaction between the *RAD51B* SNPs associated with treatment response (namely rs193127299 in perfect LD with rs140825531) and the RA susceptibility SNPs (Supplementary Figure 8).

### RA susceptibility loci

Given the finding that *RAD51B* treatment response SNPs have chromatin interactions with RA susceptibility SNPs in the same region, we used in-house Capture-HiC data from GM12878 and Jurkat cell lines to look at all variants *p* < 1 × 10^−4^ across all analyses for any further links. We found chromatin interactions between treatment response SNPs and a further 15 loci associated with RA susceptibility (Supplementary Table 4).

### Heritability estimation

The estimated heritability for each DAS28 component is presented in Table 4. We report the 90% credible interval, the posterior mean and the standard deviation from the posterior distribution of heritability under the Bayesian model, and the REML estimate of heritability and its standard error from GCTA.

**Table 4:** Heritability analysis results

Phenotype	90% Credible Interval Bayesian model	Posterior Mean Bayesian model	REML Estimate (se) GCTA	Number of samples^a^
Δlog(ESR)	0.09–0.81	0.49	0.48 (0.23)	1463
Δ√SJC28	0.08–0.69	0.40	0.39 (0.21)	1637
Δ√TJC28	0.00–0.33	0.16	0.00 (0.20)	1637
ΔPGA	0.00–0.33	0.16	0.00 (0.21)	1626

^a^After excluding samples without 6-month follow-up from the 1752 samples that passed the GWAS QC

TNFi response based on 6-month change in ESR and 6-month change in SJC28 is heritable, with 90% of the posterior probability lying between 0.09–0.81 for ESR response and 0.08–0.69 for SJC28 response, clearly excluding 0. The posterior mean estimates are 0.49 and 0.40, for ESR response and SJC28 response, respectively. On the contrary, for TJC28 and PGA response there is no evidence of non-zero heritability, with 0 being inside the 90% credible interval. Furthermore, the posterior mode for TJC28 and PGA response is at 0, while for ESR and SJC28 response the posterior mode is at 0.53 and 0.36, respectively (Supplementary Figure 9). Results using non-transformed residuals and different GRM constructions are consistent with the results in Table 4 (Supplementary Figure 10).

## Discussion

In a large study of TNFi response in the UK, we have found no variants predictive of change in DAS28 score over the first 3–6 months of treatment. Nonetheless, stratified analysis has revealed several potentially interesting associations worthy of further investigation. First, we found that a variant in the *FTO* gene, robustly associated with BMI in several studies, was significantly associated with improvement in the SJC28 in infliximab-treated patients. The association is of particular interest as high BMI was shown to be a significant predictor of lower drug levels in both adalimumab and etanercept treated patients in BRAGGSS [29] and postulated to contribute to non-response through lack of bioavailability and from the increased inflammatory effect of adipose tissue [30]. A conclusion of the study in BRAGGSS [29] was to potentially switch high BMI patients to TNFi agents that account for patient weight. Infliximab is one such drug and is administered by intravenous infusion. Therefore, the finding here of *FTO* in infliximab treated patients, and the results after controlling for BMI, suggest an association independent of BMI. Further replication of this signal will be required and it is possible that other measures of obesity, such as hip/waist ratio, may be required to disentangle the effect.

The association of *BRINP1* gene locus variant with ΔSJC28 in adalimumab treated patients potentially links TNFi response to immune regulation. BRINP1 (DBC1) downregulation in a mouse model prevents FOXP3 degradation, maintaining CD4+ Treg function under TNF-α and IL-6 inflammatory stimuli [28]. Treg function is known to be impaired in RA [31] so it could be postulated that this variant is driving a positive response to adalimumab through a down-regulation of *DBC1*, thereby boosting Treg function. This supports the finding that adalimumab, but not etanercept, can expand functional Treg cells in RA [32].

It is interesting to note that interactions between TNF-response associated variants are observed with 16 RA susceptibility variants, including the *RAD51B*, *TNFAIP3*, and *PTPRC* genes. The latter has now been implicated as a predictor of TNFi response in multiple studies [33–35], whilst other studies have reported no association [36, 37]. However, it should be noted that the fragment sizes for the Capture Hi-C experiments are large (~4 kb) so it is possible that the apparent enrichment is an artefact. Further work, which might include the targeting of the treatment response SNPs and repeating the capture Hi-C experiments, will be required to ascertain the mechanisms underlying these interactions.

In the current study it is interesting to note that our most significant findings were when subcomponents of the DAS28 score were analysed separately, particularly the change in SJC28. We have previously argued that genetic changes are likely to affect a biological process, which are more likely to be captured by objective rather than subjective measures. This is supported by the heritability analysis results where we demonstrated that TNFi response as measured by 6-month change in the objective components of the DAS28 (ESR and SJC28) are heritable. The low evidence for heritability of the subjective DAS28 components (TJC28 and PGA) is in line with other studies that have found that these components of the score do not correlate with synovitis assessed by magnetic resonance imaging [38]. This is not to suggest patient attitudes to treatment and their well-being should be ignored, as this is likely to impact adherence. Furthermore, we cannot exclude a heritable component to TJC28 and PGA, albeit smaller than that of SJC28 and ESR, that the study was underpowered to detect. Indeed, a previous study in a Dutch population reported a higher heritability for TJC28 compared with the current study [39].

Whilst the study has several strengths including recruitment from a single country, a unified study design and large sample size, there are also several limitations that should be recognised. First, we have previously reported in a study of 748 patients, from the same BRAGGSS cohort, that 27% of patients self-reported non-adherence to TNFi therapy in the first 6 months of treatment [40]. Therefore, as with all treatment response studies, adherence to treatment is a potential confounder, which we have not been able to account for in the current work because the data were not available for all patients. This is less of a problem for infliximab, where administration is by intravenous infusion in a clinical setting.

Second, we have also reported previously that immunogenicity, the development of antidrug antibodies (ADAbs), towards TNFi agents is also a potential confounder. In a cohort of 115 BRAGGSS patients treated with certolizumab pegol, ADAbs were detected in 37% of patients by 12 months of treatment [41]. In a similar study, the figure for adalimumab was 24.8%, but in etanercept no ADAbs were detected [29]. Work is already underway to test ADAbs and drug levels in serum samples from this cohort, which will inform future research, but it was not possible to adjust for this in the current analysis.

The lack of strong genetic effects in our GWAS suggests that anti-TNF response is highly polygenic with many small effects. The sample size required to detect associations with SNPs, or to construct a useful genetic predictor from associations detected in a GWAS, may be larger than is feasible for studies of drug response. Alternative approaches to constructing genetic predictors, such as genetic scores for related traits or biomarkers using summary results from large GWAS, may have better utility.

In conclusion, we have demonstrated that TNFi response as measured by 6-month change in the objective components of the DAS28 (ESR and SJC28) is heritable, and thus genetic stratification of patients could improve targeting of treatment. We have found a number of potentially interesting variants associated with response to different TNFi agents. Many of these variants have demonstrated chromatin interactions to strong candidate genes in relevant cell types. Future studies will be necessary to determine the function of these SNPs but such interactions could suggest a potential regulatory function.

## Electronic supplementary material

Supplementary Tables & Figures

Supplementary Information 1
