# Supplementary material for: Genome-wide association study of response to tumour necrosis factor inhibitor therapy in rheumatoid arthritis
Source: Pharmacogenomics J. 2018 Aug 31;18(5):657–64. doi: 10.1038/s41397-018-0040-6 (PMC6150911; doi:10.1038/s41397-018-0040-6)
Supplement: Supplementary file 1 — Supplementary Tables & Figures [file 41397_2018_40_MOESM1_ESM.pdf]

Supplementary Table 1

The number of samples genotyped and the genotyping arrays used for the current study

Imputation group	n	Number of SNPs
Affymetrix group	**825**	272 006
Affymetrix_5/6	577	
Affymetrix_6	248	
Illumina Omni-HumanCore group	**834**	204 894
Illumina_CoreExome	330	
Illumina_OmniExpress	504	
Illumina HumanHap group	**111**	460 812
Illumina_550	41	
Illumina_550/650	70	
**Total**	**1770**	

Supplementary Table 2

Association analysis results ($p < 1x\,10^{-7}$) for variants with MAF ≤ 5% but ≥ 1%

Pheno	TNFi	Gene region	SNP	Chr	BP	A1	A2	Genotype counts	MAF	P	Beta	SE
**MAF < 5%**												
SJC28	All	intronic CSMD3	rs150537045	8	114407100	G	A	1669/54/0	0.016	1.71E-08	-0.50	0.09
SJC28	Infliximab	intronic RAD51B	rs140825531	14	68488674	G	A	425/9/0	0.010	6.09E-10	-1.44	0.23
SJC28	Infliximab	intergenic LOC286114/LOC101929172	rs10097964	8	20982903	G	A	425/9/0	0.010	3.80E-08	-1.28	0.23
SJC28	Infliximab	intergenic CADM2/VGLL3	rs114456288	3	86497394	G	T	425/8/1	0.012	5.29E-08	-1.10	0.20
SJC28	Etanercept	intergenic ADGRL4/LOC101927412	rs76668869	1	80979421	G	C	598/18/3	0.019	1.60E-11	-0.75	0.11
SJC28	Etanercept	intergenic FAR1/SPON1	rs78078856	11	13758792	T	A	605/14/0	0.011	1.57E-08	-0.91	0.16
SJC28	Adalimumab	intronic KIAA0391	rs2295463	14	35736273	C	T	534/17/0	0.015	3.18E-08	-0.84	0.15
SJC28	Adalimumab	intronic DLG2	rs117214796	11	84871677	T	C	535/16/0	0.015	3.50E-08	-0.86	0.16
SJC28	Adalimumab	intronic NEBL	rs74807419	10	21340093	C	G	534/14/3	0.018	5.99E-08	-0.66	0.12
SJC28	Adalimumab	intronic EMCN	rs34619498	4	101426732	T	C	523/28/0	0.025	7.92E-08	-0.64	0.12
ESR	Adalimumab	intergenic LINC00845/TMEM26	rs140142800	10	62796237	C	T	464/23/0	0.024	6.18E-08	-0.79	0.15
TJC28	Etanercept	intronic NAV2	rs78368496	11	19984329	A	G	586/32/1	0.027	4.15E-08	-0.69	0.13
TJC28	Adalimumab	intergenic MIR4277/MRPL36	rs147859879	5	1729788	G	C	533/18/0	0.016	4.43E-09	-1.00	0.17
TJC28	Adalimumab	intronic GABBR2	rs337527	9	101447963	T	C	538/12/1	0.013	6.50E-08	-0.98	0.18

Supplementary Table 3

Association of all variants with $p < 1x\,10^{-7}$ in one phenotype (in **bold**), across other phenotypes where $p < 0.01$. Low frequency variants are in red.

SNP	Chr	A1	SNP location	Genotype Counts	MAF	P	Beta	Phenotype	TNFi
**rs10097964**	8	G	LOC286114,LOC101929172	425/9/0	0.010	3.80E-08	-1.28	deltasjc	infliximab
rs10097964	8	G	LOC286114,LOC101929172	425/9/0	0.010	7.12E-05	-1.07	deltatjc	infliximab
rs10097964	8	G	LOC286114,LOC101929172	432/9/0	0.010	6.05E-04	-1.11	deltadas	infliximab
rs10097964	8	G	LOC286114,LOC101929172	1677/46/0	0.013	6.44E-04	-0.33	deltasjc	all
**rs10739537**	9	G	BRINP1,LINC01613	70/275/206	0.377	9.11E-08	-0.21	deltasjc	adalimumab
rs10739537	9	G	BRINP1,LINC01613	70/275/206	0.377	2.85E-04	-0.17	deltatjc	adalimumab
**rs114456288**	3	G	CADM2,VGLL3	425/8/1	0.012	5.29E-08	-1.10	deltasjc	infliximab
rs114456288	3	G	CADM2,VGLL3	425/8/1	0.012	5.46E-04	-0.80	deltatjc	infliximab
rs114456288	3	G	CADM2,VGLL3	1669/39/1	0.012	2.80E-03	-0.39	deltapga	all
rs114456288	3	G	CADM2,VGLL3	417/8/1	0.012	4.70E-03	-0.69	deltapga	infliximab
rs114456288	3	G	CADM2,VGLL3	431/9/1	0.012	4.98E-03	-0.76	deltadas	infliximab
rs114456288	3	G	CADM2,VGLL3	537/12/0	0.011	9.57E-03	0.62	deltaesr	etanercept
**rs11599217**	10	G	C10orf90,DOCK1	128/210/96	0.463	7.27E-08	-0.29	deltatjc	infliximab
rs11599217	10	G	C10orf90,DOCK1	130/214/97	0.463	5.04E-06	-0.29	deltadas	infliximab
rs11599217	10	G	C10orf90,DOCK1	126/206/94	0.462	2.14E-03	-0.17	deltapga	infliximab
rs11599217	10	G	C10orf90,DOCK1	128/210/96	0.463	7.14E-03	-0.13	deltasjc	infliximab
rs11599217	10	G	C10orf90,DOCK1	417/791/306	0.463	8.60E-03	-0.08	deltaesr	all
**rs117214796**	11	T	DLG2	535/16/0	0.015	3.50E-08	-0.86	deltasjc	adalimumab
rs117214796	11	T	DLG2	535/16/0	0.015	4.80E-05	-0.74	deltatjc	adalimumab
rs117214796	11	T	DLG2	1687/36/0	0.010	3.36E-04	-0.39	deltasjc	all
rs117214796	11	T	DLG2	1687/36/0	0.010	4.24E-03	-0.36	deltatjc	all
**rs140142800**	10	C	LINC00845,TMEM26	464/23/0	0.024	6.18E-08	-0.79	deltaesr	adalimumab
rs140142800	10	C	LINC00845,TMEM26	476/24/0	0.024	3.99E-03	-0.53	deltadas	adalimumab
rs140142800	10	C	LINC00845,TMEM26	1433/80/1	0.027	4.11E-03	-0.26	deltaesr	all
rs140142800	10	C	LINC00845,TMEM26	522/29/0	0.026	8.85E-03	-0.31	deltasjc	adalimumab
**rs140825531**	14	G	RAD51B	425/9/0	0.010	6.09E-10	-1.44	deltasjc	infliximab
rs140825531	14	G	RAD51B	1670/53/0	0.015	9.27E-05	-0.35	deltasjc	all
rs140825531	14	G	RAD51B	1522/47/0	0.015	1.11E-04	-0.53	deltadas	all
rs140825531	14	G	RAD51B	1658/51/0	0.015	1.14E-03	-0.39	deltapga	all
rs140825531	14	G	RAD51B	1670/53/0	0.015	3.51E-03	-0.31	deltatjc	all
rs140825531	14	G	RAD51B	417/9/0	0.011	4.01E-03	-0.81	deltapga	infliximab
rs140825531	14	G	RAD51B	432/9/0	0.010	6.33E-03	-0.88	deltadas	infliximab
rs140825531	14	G	RAD51B	480/20/0	0.020	9.31E-03	-0.52	deltadas	adalimumab
**rs147859879**	5	G	MIR4277,MRPL36	533/18/0	0.016	4.43E-09	-1.00	deltatjc	adalimumab
rs147859879	5	G	MIR4277,MRPL36	533/18/0	0.016	1.60E-05	-0.63	deltasjc	adalimumab
rs147859879	5	G	MIR4277,MRPL36	484/16/0	0.016	5.57E-05	-0.90	deltadas	adalimumab
rs147859879	5	G	MIR4277,MRPL36	1680/43/0	0.012	1.35E-03	-0.32	deltasjc	all
rs147859879	5	G	MIR4277,MRPL36	531/18/0	0.016	3.30E-03	-0.59	deltapga	adalimumab
rs147859879	5	G	MIR4277,MRPL36	1680/43/0	0.012	5.04E-03	-0.33	deltatjc	all
rs147859879	5	G	MIR4277,MRPL36	1531/38/0	0.012	7.16E-03	-0.41	deltadas	all
**rs150537045**	8	G	CSMD3	1669/54/0	0.016	1.71E-08	-0.50	deltasjc	all
rs150537045	8	G	CSMD3	526/25/0	0.023	7.89E-06	-0.56	deltasjc	adalimumab
rs150537045	8	G	CSMD3	526/25/0	0.023	1.88E-04	-0.55	deltatjc	adalimumab

Continued...

Supplementary Table 3 continued

SNP	Chr	A1	SNP location	Genotype Counts	MAF	P	Beta	Phenotype	TNFi
rs150537045	8	G	CSMD3	420/14/0	0.016	1.00E-03	-0.62	deltasjc	infliximab
rs150537045	8	G	CSMD3	478/22/0	0.022	1.53E-03	-0.61	deltadas	adalimumab
rs150537045	8	G	CSMD3	1669/54/0	0.016	1.58E-03	-0.33	deltatjc	all
rs150537045	8	G	CSMD3	1517/52/0	0.017	2.19E-03	-0.40	deltadas	all
**rs2187874**	4	G	ZNF595,ZNF718	1279/397/47	0.142	7.00E-08	-0.19	deltatjc	all
rs2187874	4	G	ZNF595,ZNF718	1279/397/47	0.142	4.51E-05	-0.12	deltasjc	all
rs2187874	4	G	ZNF595,ZNF718	1163/363/43	0.143	1.26E-04	-0.18	deltadas	all
rs2187874	4	G	ZNF595,ZNF718	464/135/20	0.141	5.09E-04	-0.20	deltatjc	etanercept
rs2187874	4	G	ZNF595,ZNF718	313/105/16	0.158	3.02E-03	-0.21	deltatjc	infliximab
rs2187874	4	G	ZNF595,ZNF718	313/105/16	0.158	6.68E-03	-0.17	deltasjc	infliximab
rs2187874	4	G	ZNF595,ZNF718	1271/391/47	0.142	6.85E-03	-0.11	deltapga	all
**rs2295463**	14	C	KIAA0391	534/17/0	0.015	3.18E-08	-0.84	deltasjc	adalimumab
rs2295463	14	C	KIAA0391	485/15/0	0.015	1.70E-05	-0.99	deltadas	adalimumab
rs2295463	14	C	KIAA0391	534/17/0	0.015	6.95E-04	-0.60	deltatjc	adalimumab
rs2295463	14	C	KIAA0391	532/17/0	0.015	7.23E-03	-0.55	deltapga	adalimumab
**rs337527**	9	T	GABBR2	538/12/1	0.013	6.50E-08	-0.98	deltatjc	adalimumab
rs337527	9	T	GABBR2	488/11/1	0.013	2.46E-05	-0.97	deltadas	adalimumab
rs337527	9	T	GABBR2	538/12/1	0.013	3.98E-04	-0.55	deltasjc	adalimumab
**rs34619498**	4	T	EMCN	523/28/0	0.025	7.92E-08	-0.64	deltasjc	adalimumab
**rs7195994**	16	G	FTO	363/68/3	0.085	9.74E-09	-0.48	deltasjc	infliximab
rs7195994	16	G	FTO	369/69/3	0.085	4.28E-05	-0.48	deltadas	infliximab
rs7195994	16	G	FTO	363/68/3	0.085	1.01E-04	-0.38	deltatjc	infliximab
rs7195994	16	G	FTO	1290/269/10	0.092	9.39E-04	-0.19	deltadas	all
rs7195994	16	G	FTO	1414/297/12	0.093	1.89E-03	-0.14	deltatjc	all
rs7195994	16	G	FTO	1414/297/12	0.093	2.25E-03	-0.12	deltasjc	all
**rs74807419**	10	C	NEBL	534/14/3	0.018	5.99E-08	-0.66	deltasjc	adalimumab
rs74807419	10	C	NEBL	534/14/3	0.018	5.74E-05	-0.57	deltatjc	adalimumab
rs74807419	10	C	NEBL	1672/47/4	0.016	7.54E-04	-0.28	deltasjc	all
rs74807419	10	C	NEBL	1672/47/4	0.016	4.66E-03	-0.27	deltatjc	all
**rs76668869**	1	G	ADGRL4,LOC101927412	598/18/3	0.019	1.60E-11	-0.75	deltasjc	etanercept
rs76668869	1	G	ADGRL4,LOC101927412	598/18/3	0.019	7.59E-06	-0.61	deltatjc	etanercept
rs76668869	1	G	ADGRL4,LOC101927412	543/18/3	0.021	2.01E-04	-0.63	deltadas	etanercept
rs76668869	1	G	ADGRL4,LOC101927412	1639/79/5	0.026	1.82E-03	-0.21	deltasjc	all
**rs78078856**	11	T	FAR1,SPON1	605/14/0	0.011	1.57E-08	-0.91	deltasjc	etanercept
rs78078856	11	T	FAR1,SPON1	605/14/0	0.011	6.85E-06	-0.90	deltatjc	etanercept
rs78078856	11	T	FAR1,SPON1	550/14/0	0.012	1.59E-03	-0.78	deltadas	etanercept
rs78078856	11	T	FAR1,SPON1	1682/41/0	0.012	2.27E-03	-0.31	deltasjc	all
rs78078856	11	T	FAR1,SPON1	1682/41/0	0.012	3.07E-03	-0.35	deltatjc	all
**rs78368496**	11	A	NAV2	586/32/1	0.027	4.15E-08	-0.69	deltatjc	etanercept
rs78368496	11	A	NAV2	586/32/1	0.027	1.01E-03	-0.34	deltasjc	etanercept
rs78368496	11	A	NAV2	1594/126/3	0.038	6.31E-03	-0.18	deltatjc	all
**rs948138**	11	G	MMP20,MMP27	132/292/191	0.452	7.62E-08	0.25	deltapga	etanercept
rs948138	11	G	MMP20,MMP27	115/272/177	0.445	6.56E-04	0.18	deltadas	etanercept
rs948138	11	G	MMP20,MMP27	342/849/518	0.449	2.13E-03	0.09	deltapga	all

Supplementary Table 4

Chromatin interactions between treatment response SNPs and RA susceptibility SNPs. (*) indicates promoter as well as region capture in that cell type, (**) indicates promoter capture only. Low frequency variants are in red.

Phenotype	C-HiC data	RA locus SNPs	Chr	Best P	SNP	Minor allele frequency	Best Interaction score
DAS28-ESR infliximab	GM* & Jurkat	*ANXA3*	4	6.00E-05	rs1061400	0.170	7.75
ESR infliximab	GM* & Jurkat	*ANXA3*	4	9.44E-05	rs1061400	0.167	7.75
DAS28-ESR infliximab	GM**	*BLK*	8	4.49E-05	rs77314514	0.048	6.81
ESR infliximab	GM* & Jurkat**	*BLK*	8	4.57E-05	rs2645453	0.421	12.71
ESR All	GM* & Jurkat**	*BLK*	8	6.21E-05	rs2645453	0.427	12.71
SJC28 adalimumab	GM*	*C4orf52*	4	8.23E-05	rs116577984	0.012	15.68
ESR infliximab	Jurkat	*EOMES*	3	4.38E-05	rs114990163	0.017	6.40
TJC28 adalimumab	GM	*EOMES*	3	5.16E-05	rs35112923	0.025	6.89
PGA adalimumab	Jurkat	*EOMES*	3	9.31E-05	rs114178819	0.019	5.07
ESR adalimumab	GM & Jurkat	*IFNGR2*	21	8.71E-05	rs139387252	0.016	9.73
ESR All	GM & Jurkat	*IFNGR2*	21	8.89E-05	rs139387252	0.018	9.73
ESR adalimumab	Jurkat**	*MED1*	17	7.46E-06	rs72823322	0.085	5.23
DAS28-ESR All	GM* & Jurkat	*PADI4*	1	8.10E-05	rs58218115	0.194	9.00
DAS28-ESR adalimumab	GM	*PRKCH*	14	5.07E-06	rs45544631	0.016	6.09
TJC28 adalimumab	GM	*PRKCH*	14	1.65E-05	rs45544631	0.016	6.09
DAS28-ESR etanercept	GM* & Jurkat	*PTPRC*	1	3.40E-05	rs77622698	0.035	14.04
SJC28 All	GM	*RAD51B*	14	9.01E-05	rs193127299	0.016	11.15
SJC28 infliximab	GM	*RAD51B*	14	6.09E-10	rs193127299	0.010	11.15
TJC28 adalimumab	GM & Jurkat	*RAD51B*	14	4.48E-05	rs17835200	0.058	5.69
PGA All	GM & Jurkat	*RAD51B*	14	1.25E-05	rs117314383	0.030	6.29
DAS28-ESR All	GM & Jurkat	*RAD51B*	14	1.90E-05	rs117314383	0.030	22.66
SJC28 etanercept	GM**	*RCAN1*	21	4.34E-06	rs117641308	0.017	6.76
TJC28 adalimumab	GM & Jurkat	*RUNX1*	21	2.83E-06	rs13047746	0.042	29.51
SJC28 All	GM	*SPRED2*	2	5.71E-05	rs897875	0.037	5.53
DAS28-ESR All	GM* & Jurkat*	*TNFAIP3*	6	8.57E-05	rs112862764	0.09	6.03
DAS28-ESR All	GM* & Jurkat*	*TNFAIP3*	6	8.24E-05	rs4896297	0.088	8.70
ESR adalimumab	GM	*TNFRSF9*	1	3.24E-05	rs2797685	0.173	5.62
DAS28-ESR etanercept	GM**	*TRAF1-C5*	9	8.90E-05	rs3762054	0.249	5.70

Supplementary Figure 1

Histograms showing the raw distribution of ΔSJC28, ΔTJC28, ΔESR, ΔPGA, and ΔDAS28. Note: the values were mean-centred and scaled to have a variance of 1 before linear regression (see Methods).

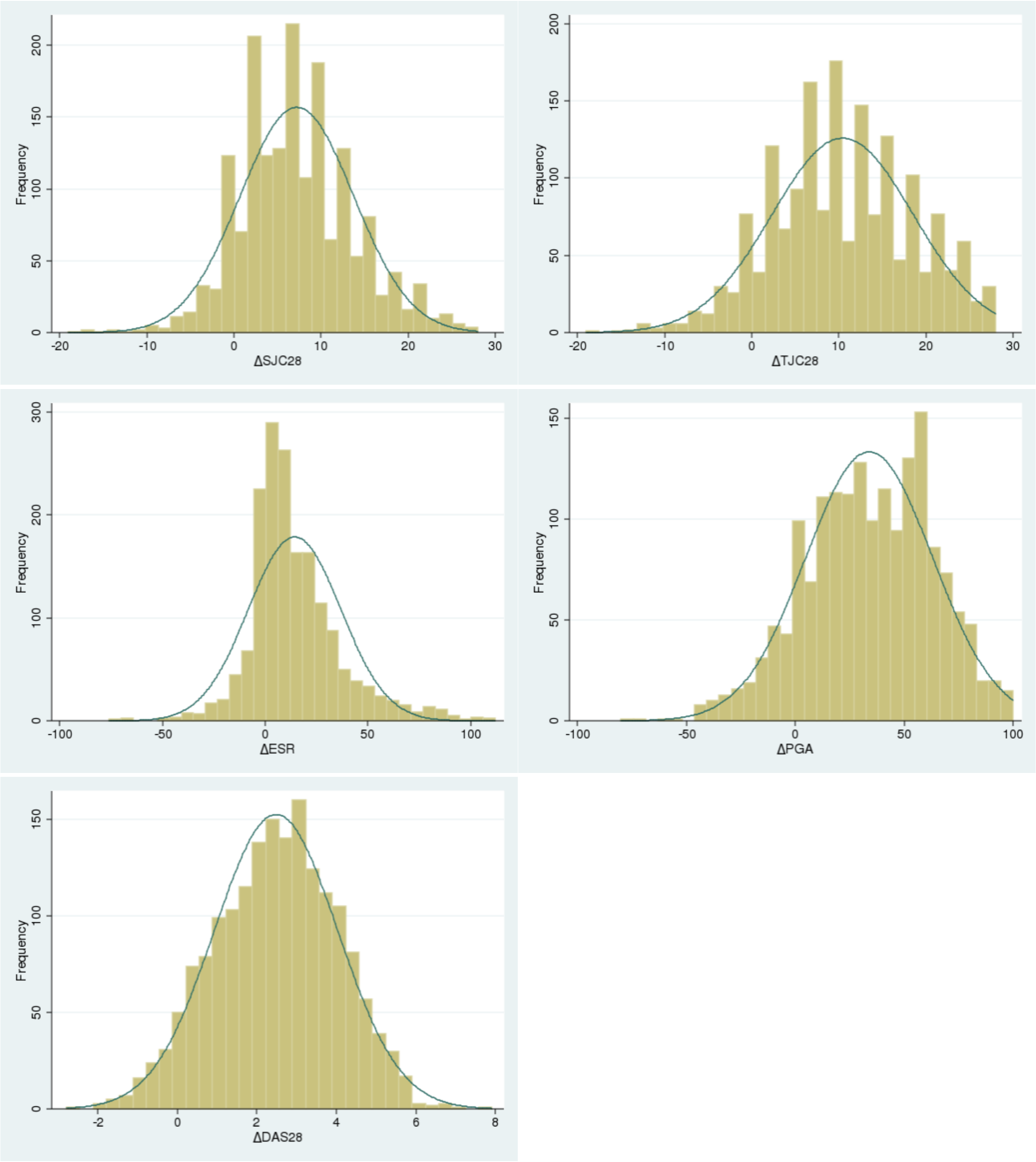

Supplementary Figure 2

The Quantile-Quantile (QQ) plots for each analysis (A=SJC28, B=TJC28, C=ESR, D=PGA, E=DAS28)

A

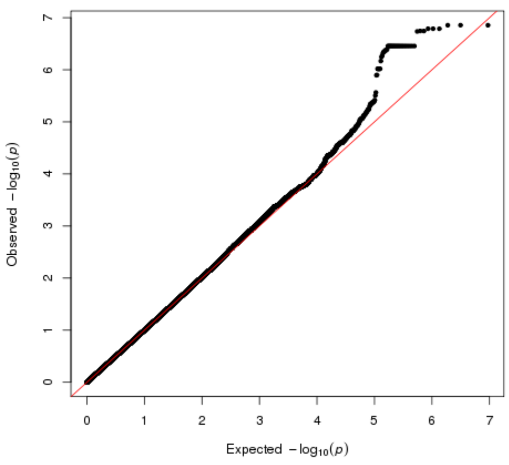

B

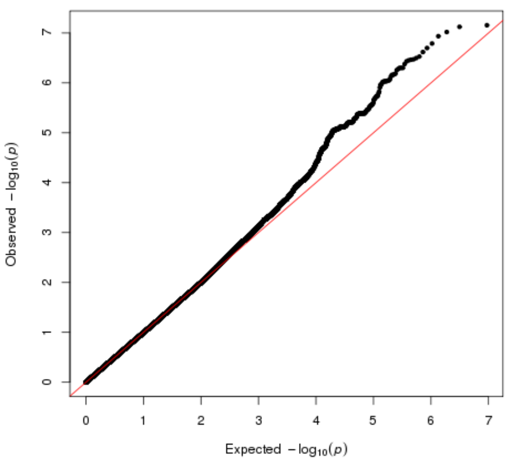

C

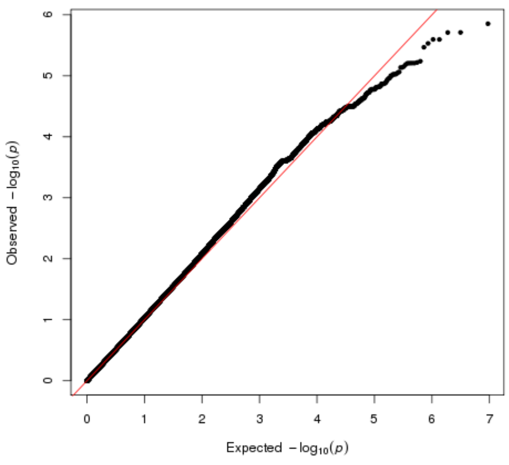

D

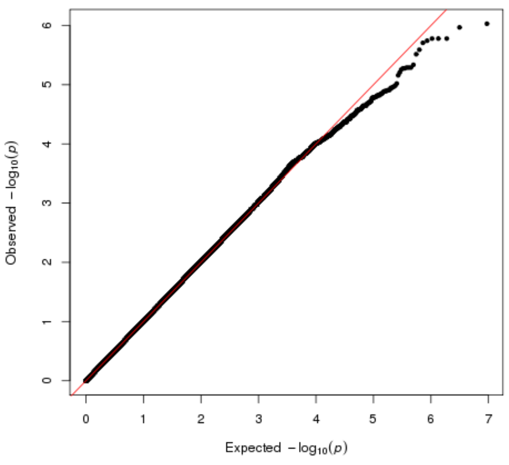

E

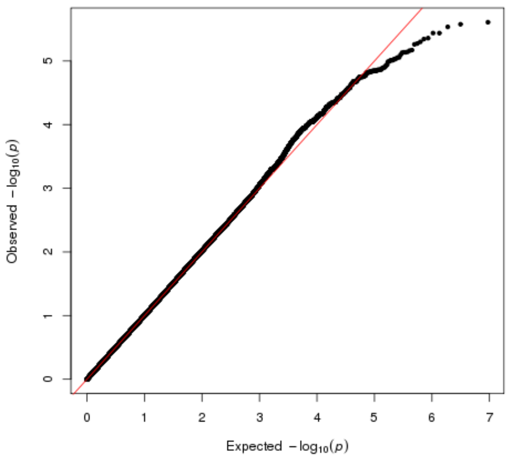

Supplementary Figure 3

A Regional association plot for rs2187874 [purple diamond]

Circles represent SNPs in LD, with the colour representing the strength of LD as measured by r^2 (see key). Genes are shown as horizontal lines at the bottom of the plot, with chromosomal position shown in Mb.

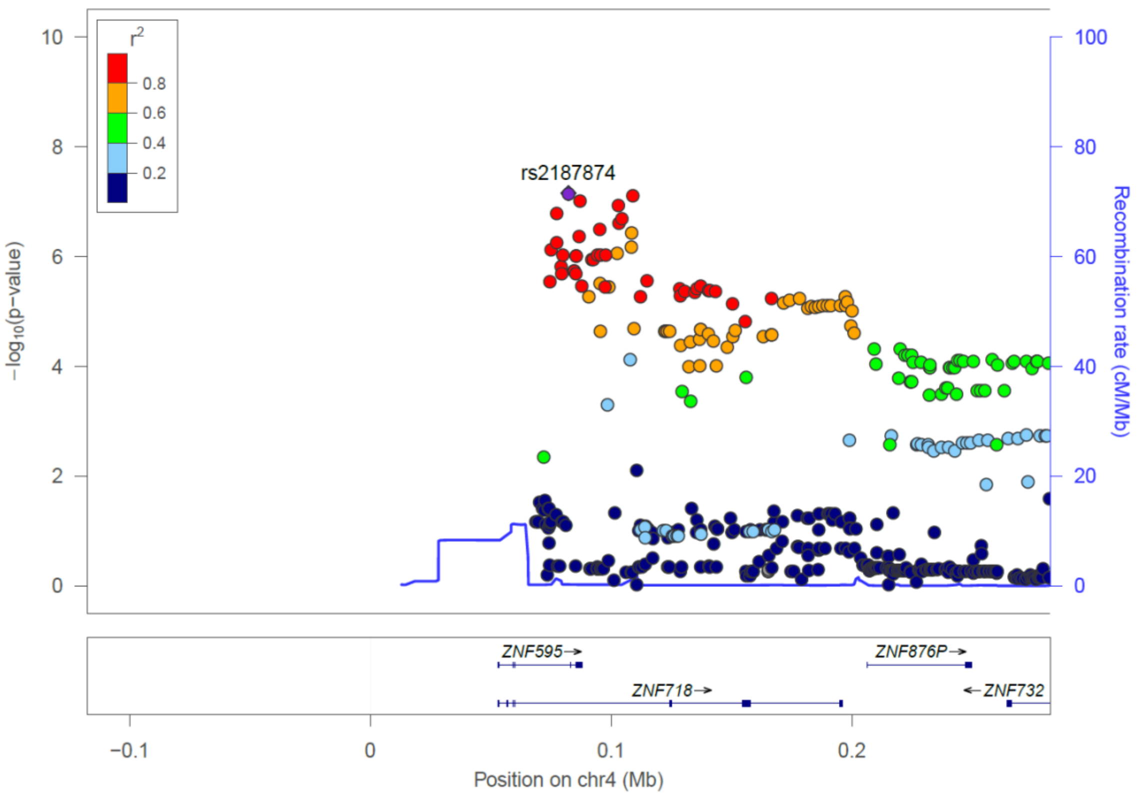

B Chromatin interactions in GM12878 cells with rs2187874 [in red]

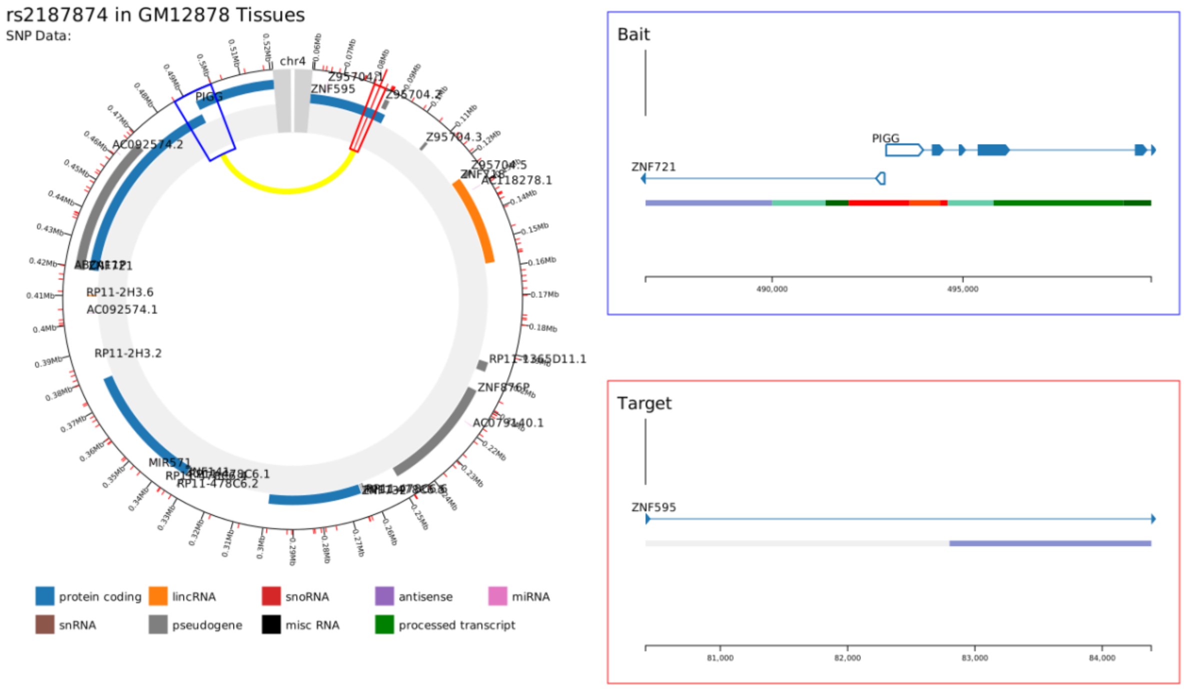

Supplementary Figure 4

A Regional association plot for rs7195994

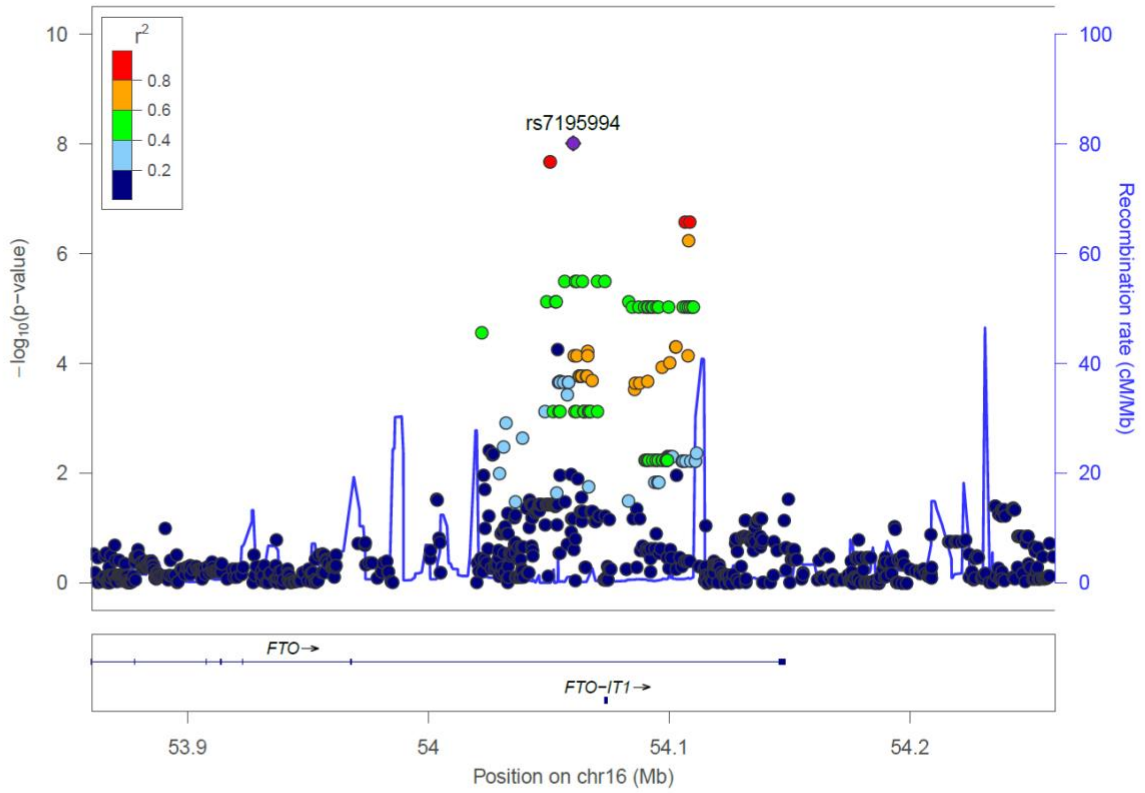

B Chromatin interactions in GM12878 cells with rs2540767 [in red] (a SNP in LD with above)

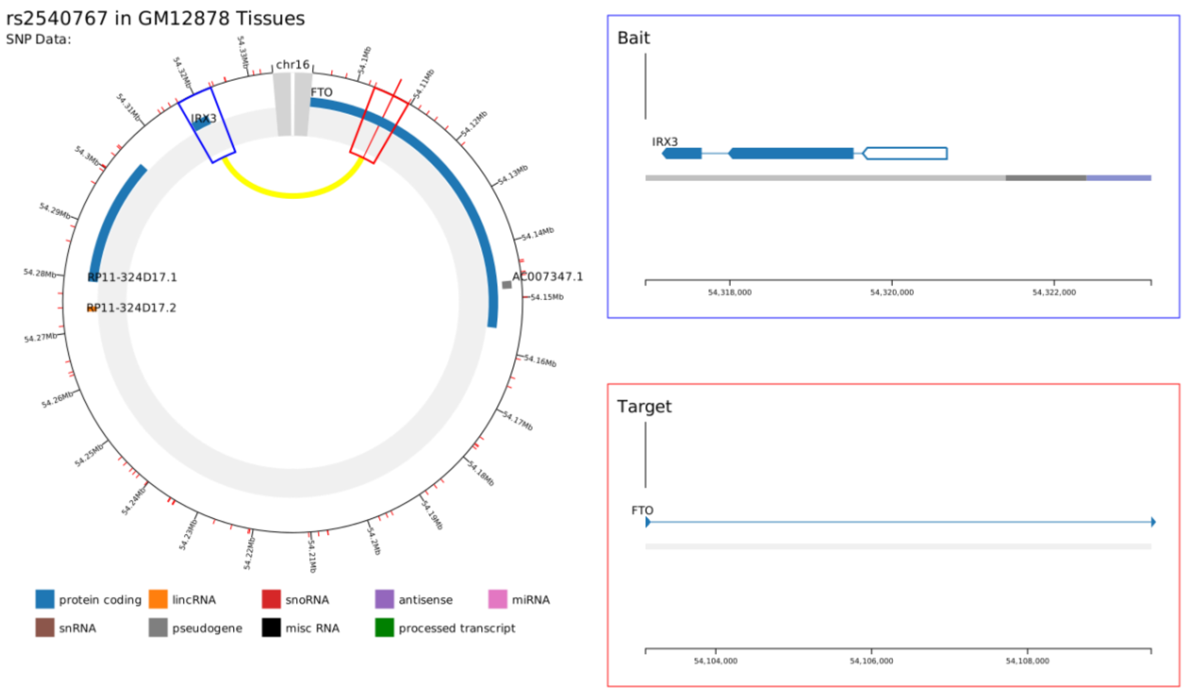

Supplementary Figure 5

Regional association plot for rs11599217

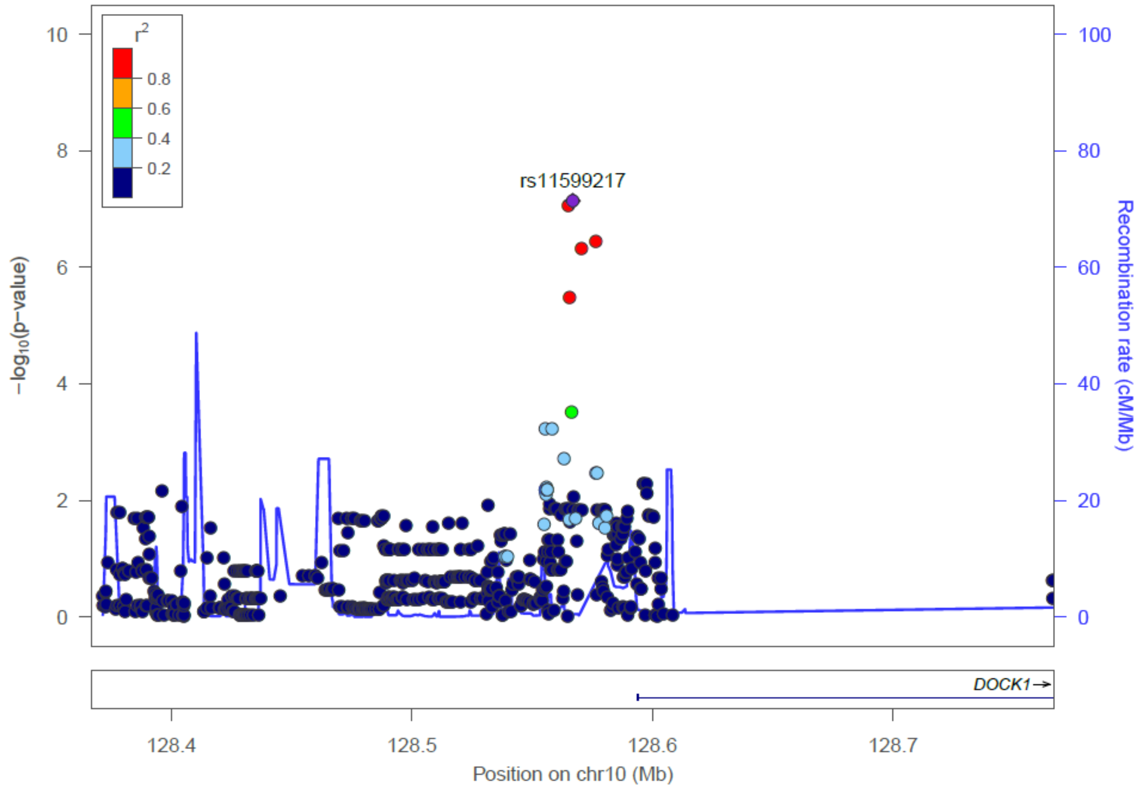

Supplementary Figure 6

Regional association plot for rs10739537

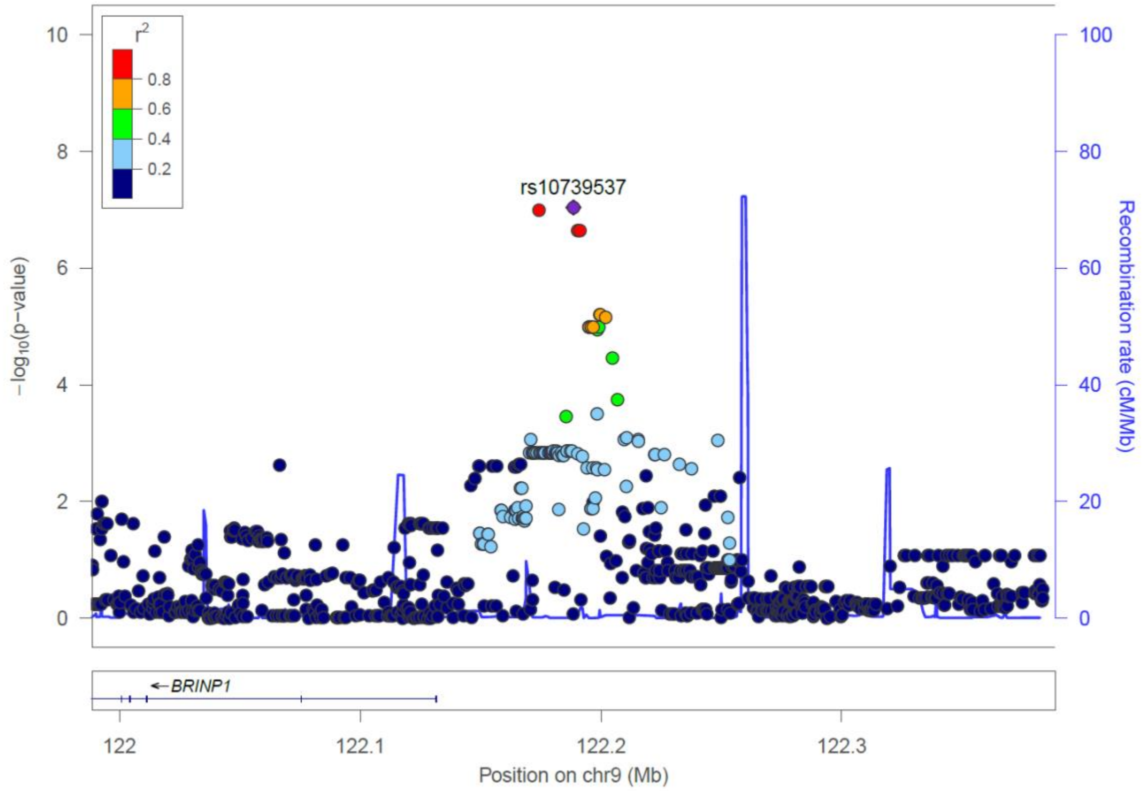

Supplementary Figure 7

A Regional association plot for rs948138

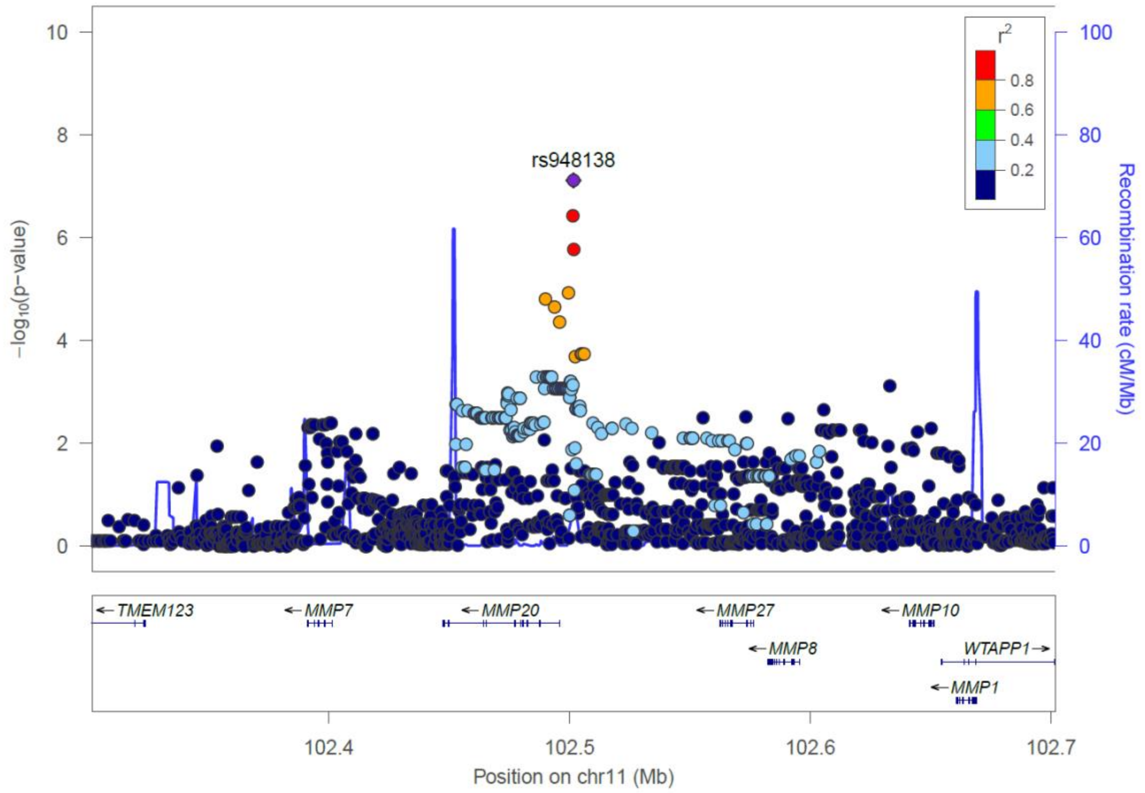

B Chromatin interactions in Naive CD8 cells with rs948138 [in red]

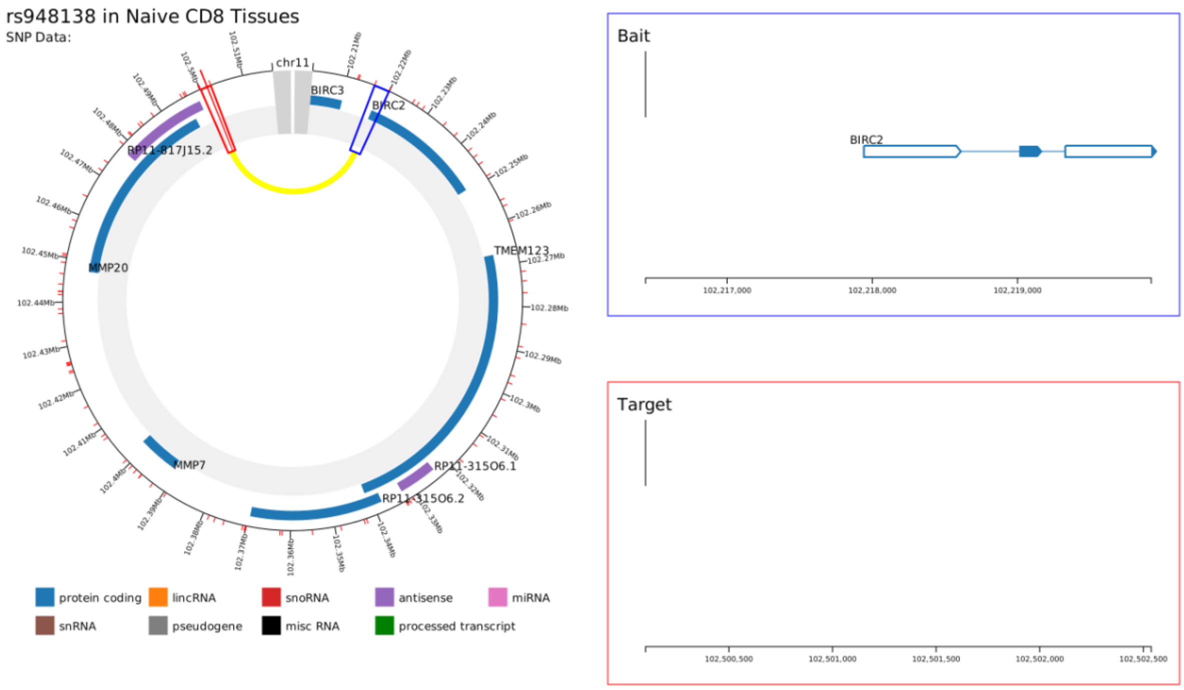

Supplementary Figure 8

Capture HiC in-house data. Interaction (blue arc) is shown between the HindIII fragment containing the rs193127299 SNP (red line) and the RA susceptibility/risk SNPs (green).

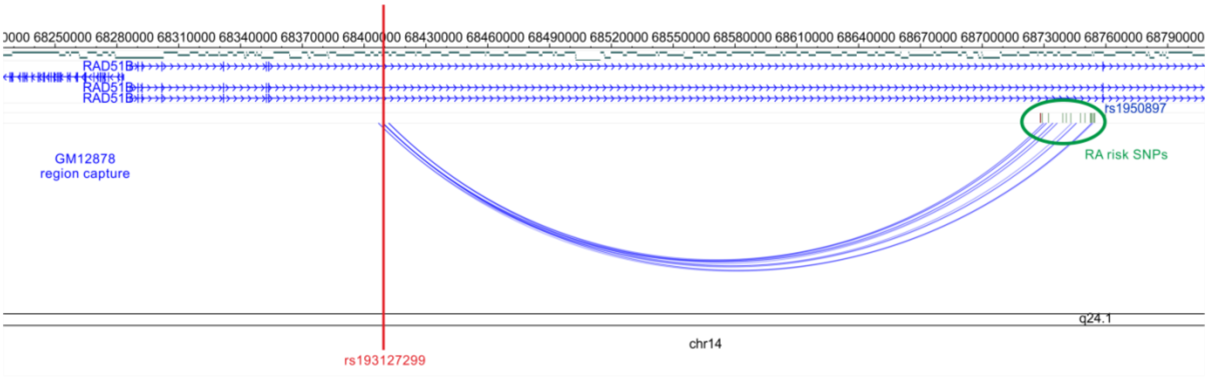

Supplementary Figure 9

Posterior densities for the heritability of the DAS28 components

TNFi response is measured by the rank-transformed residuals of the 6-month change in each DAS component. The Genetic Relationship Matrix (GRM) is computed by taking the inner product between the vectors of genotypes for each pair of individuals, normalised to unit vectors. ESR and SJC28 response are heritable, with the 90% credible interval excluding 0. On the contrary, there is no evidence of nonzero heritability for TJC28 and PGA (VAS) response with the posterior density being concentrated towards 0.

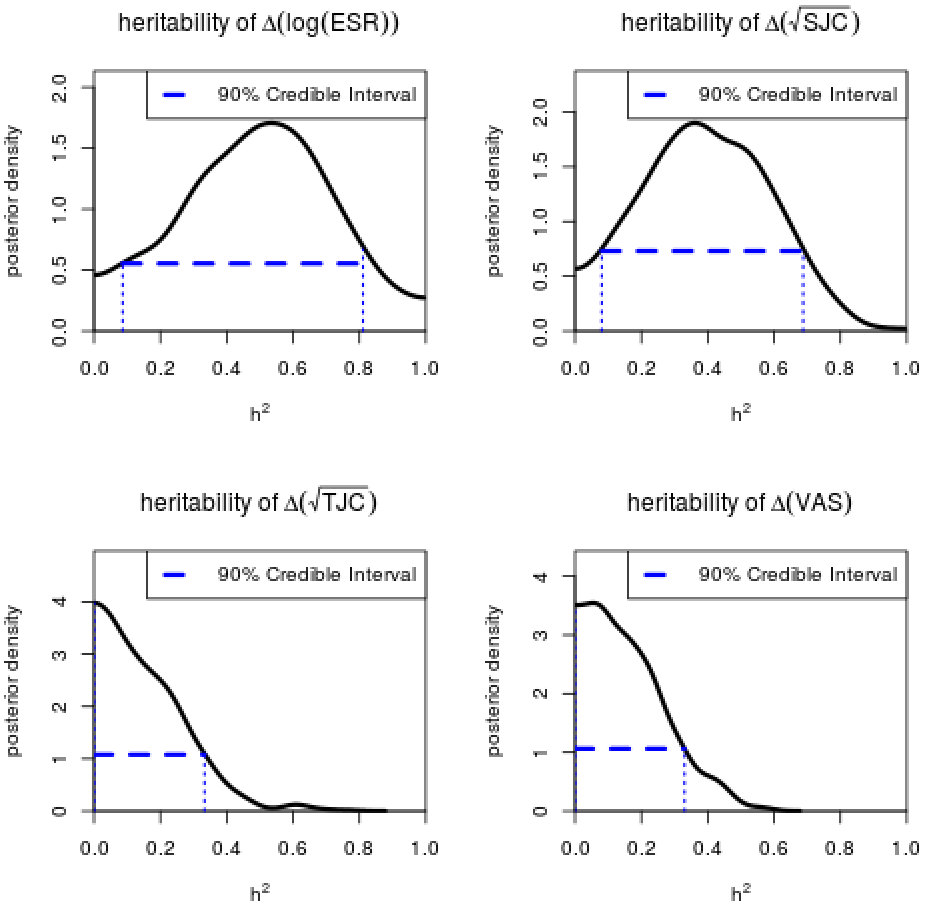

Supplementary Figure 10

Comparison of GRM constructions and outcome transformations in estimating heritability of TNFi response. The middle of each box corresponds to the posterior mean for the Bayesian model and to the REML point estimate for GCTA. The top and bottom of the box correspond to the middle plus/minus one standard deviation for the Bayesian model and plus/minus one standard error for GCTA. For the Bayesian model, the whiskers correspond to the 90% credible interval.

“p0” denotes a GRM without pre-scaling of SNPs to unit variance, while “p1” denotes a GRM with pre-scaling. The former construction assumes that SNP effect sizes are independent of the minor allele frequency, while the latter construction assumes that rarer SNPs have larger effect sizes and is the default construction in GCTA. The grouping corresponds to un-transformed (“no”) and rank-transformed (“rank”) residuals of the 6-month change.

Results from the Bayesian model are consistent with those from GCTA. The effect of the rank-transformation is small. On the other hand, the GRM construction is important. Scaling of the SNPs to unit variance (“p1”) leads to higher estimates for heritability. In the main text we report the more conservative estimates based on the “p0” GRM construction.

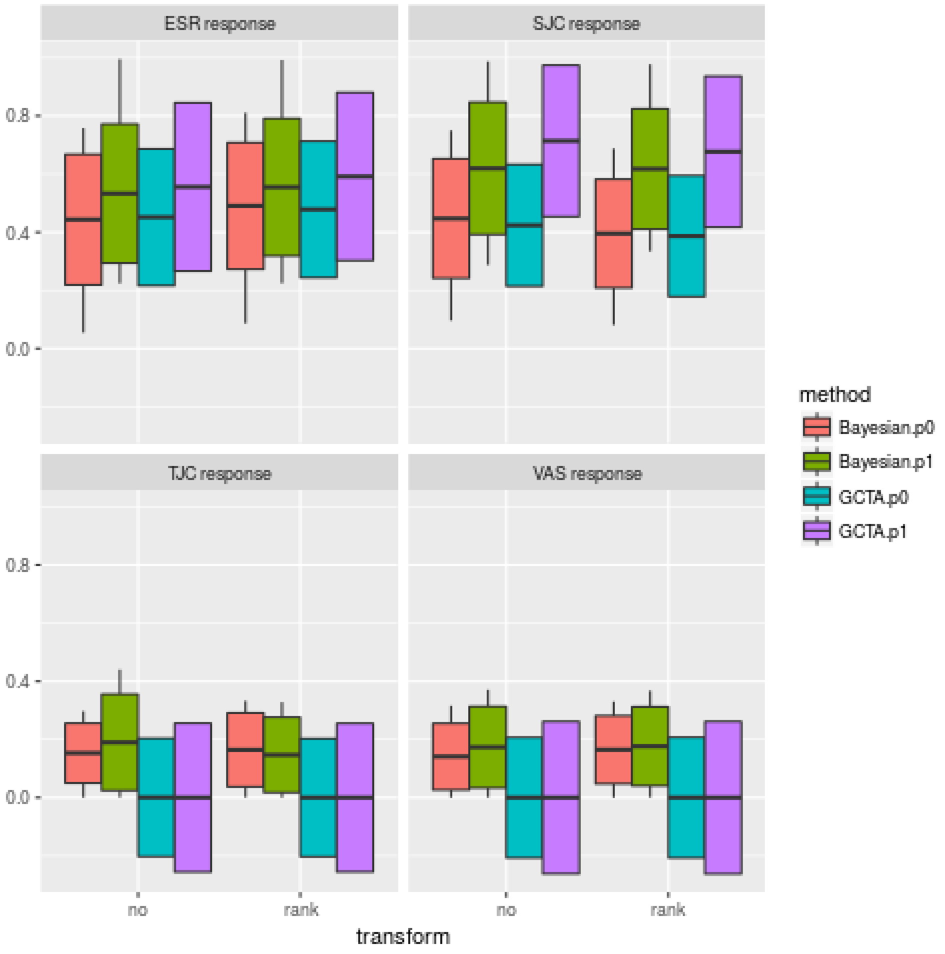

Members of the Biologics in Rheumatoid Arthritis Genetics and Genomics Study Syndicate (BRAGGSS)

Steering Committee members: Professor Anne Barton, Professor John Isaacs, Professor Ann Morgan, Professor Gerry Wilson

Barts Health NHS Trust (Prof C Pitzalis)

Basingstoke & North Hampshire NHS Foundation Trust (Dr E Williams, Dr R K Moitra, Dr D J Shawe)

Burton Hospitals NHS Foundation Trust (Dr M Nisar)

Central Manchester University Hospitals NHS Foundation Trust

- **Manchester Royal Infirmary** (Prof I Bruce, Prof A Barton, Dr R Gorodkin, Dr P Ho, Dr K Hyrich)
- **Trafford General** (Dr F McKenna)

Chesterfield Royal Hospital NHS Foundation Trust (Dr K Fairburn)

Countess of Chester Hospital NHS Foundation Trust (Dr J Nixon, Dr T Barnes, Dr M Hui)

City Hospitals Sunderland NHS Foundation Trust (Dr D Coady, Dr D Wright, Dr C Morley, Dr G Raftery, Dr C Bracewell)

County Durham and Darlington NHS Foundation Trust (Dr R Reece, Dr. D. Armstrong, Dr. A J Chuck, Dr. S Hailwood, Dr N Kumar, Dr D Ashok,)

Derby Hospitals NHS Foundation Trust (Dr. S C O'Reilly, Dr T Ding, Dr. L J Badcock, Dr. C M Deighton , Dr N Raj, Dr. M R Regan, Dr. G D Summers, Dr. R A Williams)

Gateshead Health NHS Foundation Trust (Dr. C A Kelly, Dr. J Hamilton, Dr. C R Heycock, Dr V Saravanan)

Harrogate & District NHS Foundation Trust (Dr M Green, Dr A Gough, Dr C Lawson)

Kettering General Hospital NHS Foundation Trust (Dr A Kuttikat, Dr D Parthajit, Dr E Borbas, Dr T Wazir)

Leeds Teaching Hospitals NHS Trust (Prof. P Emery, Dr. S. Bingham, Prof. A. Morgan, Prof H A Bird, Prof P G Conaghan, Dr C T Pease, Dr R J Wakefield, Prof. M Buch, Dr S Dass)

Northumbria Healthcare NHS Foundation Trust (Dr F N Birrell, Dr P R Crook)

Northern Lincolnshire and Goole Hospitals NHS Foundation Trust (Dr B Szebenyi, Dr D Bates, Dr D James, Dr T Gillott, Dr A Alvi, C Grey, J Browning

Nottingham University Hospitals NHS Trust (Dr. J F McHale, Dr. I C Gaywood, Dr. A C Jones, Dr. P Lanyon, Dr. I Pande, Prof. M Doherty, Dr. A Gupta, Dr. P A Courtney, Dr A Srikanth, Dr A Abhishek)

Pennine Acute Hospitals NHS Trust –

- **North Manchester General Hospital** (Dr L Das, Dr. M Pattrick, Dr. H N Snowden, Dr A P Bowden, Dr E E Smith, Dr P Klimiuk, Dr D J Speden)
- **Fairfield Hospital** (Dr L Das)

Portsmouth Hospitals NHS Trust (Dr. J M Ledingham , Dr. R G Hull, Dr. F McCrae, Dr. A Cooper, Dr S A Young Min, Dr Wong, Dr Shaban)

Royal Cornwall Hospitals NHS Trust (Prof A D Woolf, Dr M Davis, Dr D Hutchinson, Dr A Endean)

Royal Liverpool and Broadgreen University Hospitals NHS Trust (Dr D Mewar, Dr E J Tunn, Dr K Nelson, Dr T D Kennedy, Dr C Dubois)

Royal National Hospital for Rheumatic Diseases NHS Foundation Trust (Dr J Pauling, Dr E Korendowych, Dr T Jenkinson, Dr R Sengupta, Dr A Bhalla, Prof N McHugh, Dr W Tillett, Dr T Ahmed)

Salford Royal NHS Foundation Trust (Dr. H Chinoy, Prof T O'Neil, Prof A Herrick, Prof A Jones, Dr R Cooper, Dr W Dixon, Dr B Harrison)

Sheffield Teaching Hospitals NHS Foundation Trust (Dr. M Akil, Dr. S Till, Dr L Dunkley, Dr R Tattersall, Dr R Kilding, Dr T Tait, Dr J Maxwell, Dr K-P Kuet)

South Tees Hospitals NHS Foundation Trust (Dr. M J Plant, Dr. F Clarke, Dr. J N Fordham, Dr S Tuck, Dr S K Pathare, Dr A Paul)

South Warwickshire General Hospital NHS Trust (Dr. C P Marguerie, Dr S P Rigby, Dr N Dunn)

Staffordshire & Stoke-on-Trent Partnership NHS Trust (Dr S Hider, Dr A Menon, Dr C Dowson, Dr S Dutta, Dr S Kamath, Dr J Packham, Dr S Price, Dr E Roddy, Dr Z Paskins, Professor A. Hassell)

Stockport NHS Foundation Trust (Dr A Ismail, Dr C Filer)

St Helens and Knowsley Hospitals NHS Trust (Dr. R Abernethy, Dr A R Clewes, Dr. J K Dawson)

The Dudley Group of Hospitals NHS Foundation Trust (Prof G Kitas, Dr N Erb, Dr R Klocke, Dr A J Whallett, Dr K Douglas, Dr A Pace, Dr R Sandhu, Dr H John)

The Ipswich Hospital NHS Trust (Dr S Lane)

The Newcastle upon Tyne Hospitals NHS Foundation Trust (Prof. J D Isaacs, Prof. H Foster, Dr. B Griffiths, Dr. I Griffiths, Dr L Kay, Dr W-F Ng, Dr. P N Platt, Dr. D J Walker, Dr P Peterson, Dr A Lorenzi, Dr M Friswell, Dr B Thompson, Dr M Lee, Dr A Pratt)

The Royal Wolverhampton Hospitals NHS Trust

- **Cannock Chase Hospital** (Dr. D Mulherin, Dr. S V Chalam, , Dr. T Price, Dr. T Sheeran, Dr S Venkatachalam, Dr S Baskar)
- **New Cross Hospital** (Dr Sabrina Raizada)

University Hospital Birmingham NHS Foundation Trust (Dr A Filer, Dr. Bowman, Dr. P Jobanputra Dr. E C Rankin)

University Hospitals of Coventry and Warwickshire NHS Trust (Dr S Dubey, Dr. K Chaudhuri, Dr A Price-Forbes, Dr J Ravindran)

University Hospitals of Leicester NHS Trust (Dr A Moorthy, Dr P Sheldon, Dr W Hassan, Dr J Francis, Dr A Kinder, Dr R Neame)

University Hospitals of Morcambe Bay NHS Trust

- Royal Lancaster Hospital (Dr. M Bukhari, Dr L Ottewell, Dr Palkonyai)
- Furness General Hospital (Dr M Bukhari)

West Suffolk Hospitals NHS Trust (Dr D T O'Reilly, Dr V Rajagopal,)

Wrightington, Wigan and Leigh Hospitals NHS Foundation Trust (Dr E Gladston Chelliah)

York Teaching Hospitals NHS Foundation Trust

- **York District Hospital** (Dr M Green, Dr M Quinn, Dr A Isdale, Dr A Brown, Dr B Saleem)
- **Scarborough Hospital** (Dr Z Al-Saffar, Dr G Koduri)

Members of the MAximizing Therapeutic Utility in RA (MATURA) consortium

Name	Institution
**Work Stream 1**	
Prof Costantino Pitzalis	Queen Mary University of London
Prof Peter Taylor	University of Oxford
Prof Ernest Choy	Cardiff University
Prof Iain McInnes	University of Glasgow
Dr Mike Barnes	Queen Mary University of London
Prof John Isaacs	Newcastle University
Prof Christopher Buckley	University of Birmingham
Prof Michael Ehrenstein	University College London
Prof Peter Sasieni	Queen Mary University of London
**Work Stream 2**	
Prof Anne Barton	University of Manchester
Prof Ann Morgan	University of Leeds
Prof Gerry Wilson	University College Dublin
Prof Paul McKeigue	University of Edinburgh
Prof Heather Cordell	Newcastle University
Prof Jenny Barrett	University of Leeds
Prof Andrew Cope	Kings College London
Prof Adam Young	University of Hertfordshire
Prof Karim Raza	University of Birmingham
Prof Katherine Payne	University of Manchester
Prof Jane Worthington	University of Manchester
Prof Deborah Symmons	University of Manchester
Prof Kimme Hyrich	University of Manchester
Prof Ian Bruce	University of Manchester
**Industry**	
Martin Hodge	Pfizer
Anthony Rowe	Janssen
Jeffrey Siegel	Roche/Genentech
Michelle Mao	BGI
Richard Watts	Qiagen
Carolyn Cuff	Abbvie
David Close	MedImmune
Philippe Coterell	Avacta
Felix Agakov	Pharmatics
