# Supplementary material for: Genome-wide association study of response to tumour necrosis factor inhibitor therapy in rheumatoid arthritis
Source: Pharmacogenomics J. 2018 Aug 31;18(5):657–64. doi: 10.1038/s41397-018-0040-6 (PMC6150911; doi:10.1038/s41397-018-0040-6)
Supplement: Supplementary file 2 — Supplementary Information 1 [file 41397_2018_40_MOESM2_ESM.pdf]

Bayesian model for heritability estimation

Consider the linear mixed model used in GCTA (Equation (2) in Yang et al. (2011)):

$$\mathbf{y} = X\beta + \mathbf{g} + \epsilon, \quad \text{with } \mathrm{var}(\mathbf{y}) = A\sigma_g^2 + I\sigma_\epsilon^2, \tag{1}$$

where $\mathbf{y}$ is an $n \times 1$ vector of phenotypes, and n is the sample size; β is a vector of fixed effects; $\mathbf{g}$ is an $n \times 1$ vector of total genetic effects, with $\mathbf{g} \sim \mathcal{N}\left(0, A\sigma_g^2\right)$, and A is the $n \times n$ genetic relationship matrix (GRM) between individuals; and ϵ is an $n \times 1$ vector of residual effects, with $\epsilon \sim \mathcal{N}\left(0, I\sigma_\epsilon\right)$.

For simplicity, in the following derivations we consider a phenotype adjusted for covariates by taking the residuals of a fixed effects linear model of the phenotype on the covariates. Equation 1 can then be written as:

$$\tilde{\mathbf{y}} = \beta_0 + \mathbf{g} + \tilde{\epsilon}, \quad \text{with } \mathrm{var}(\tilde{\mathbf{y}}) = A\sigma_g^2 + I\sigma_{\tilde{\epsilon}}^2, \tag{2}$$

where $\tilde{\mathbf{y}}$ are the residuals from the regression on the covariates scaled to have unit variance, β_0 is an intercept term, and $\tilde{\epsilon}$ are the residual effects with $\tilde{\epsilon} \sim \mathcal{N}\left(0, I\sigma_{\tilde{\epsilon}}\right)$.

Let $\tau = \frac{1}{\sigma_g^2 + \sigma_{\tilde{\epsilon}}^2}$ denote the total precision of phenotype $\tilde{\mathbf{y}}$, and $h^2 = \frac{\sigma_g^2}{\sigma_g^2 + \sigma_{\tilde{\epsilon}}^2}$ denote the heritability, i.e. the proportion of total variance explained by genotypes. We rewrite the variance of $\tilde{\mathbf{y}}$ as:

$$\mathrm{var}(\tilde{\mathbf{y}}) = \frac{1}{\tau}\left[h^2 A + (1 - h^2) I\right], \tag{3}$$

and define the following hierarchical model:

$$\begin{aligned}
\beta_0 &\sim \mathcal{N}\left(0, 0.01\right) \\
h^2 &\sim \mathcal{U}\left(0, 1\right) \\
\tau &\sim \Gamma\left(10, 10\right) \\
\tilde{\mathbf{y}} &\sim \mathcal{N}\left(\beta_0, \frac{1}{\tau}\left[h^2 A + (1 - h^2) I\right]\right)
\end{aligned}$$

We can then use Markov Chain Monte Carlo sampling to infer the posterior distribution of the parameters h^2, β_0 and τ.

MCMC efficiency

At each iteration, following an update in the parameters, the inverse of matrix (3) needs to be recomputed. This makes this approach computationally intractable for moderate to large sample sizes.

Instead, we proceed as follows. We compute the eigenvalue decomposition $A = P\Lambda P^{-1}$, where Λ is a diagonal matrix containing the eigenvalues of A, and columns of P correspond to the eigenvectors of A. Given that A is real and

symmetric, its eigenvalue decomposition can always be computed. Moreover, P is an orthogonal matrix, hence $P^{-1} = P^\top$, and the decomposition simplifies to

$$A = P\Lambda P^\top. \tag{4}$$

Using (4) and noting that $I = PP^\top$, we rewrite (3) as

$$\frac{1}{\tau} P \Big[h^2 \Lambda + (1 - h^2) I \Big] P^\top,$$

and its inverse can be explicitly written as

$$\tau P \Big[h^2 \Lambda + (1 - h^2) I \Big]^{-1} P^\top. \tag{5}$$

Note that the computation of the inverse reduces to the inversion of the diagonal matrix $h^2\Lambda + (1 - h^2)I$, which is trivially achieved by inverting each element on the diagonal, operation that has complexity linear in N.

This alternative formulation requires the computation of the eigenvalue decomposition of the genetic relationship matrix, operation commonly available in most software packages (for example, it is implemented in function `eigen()` in R). This is usually implemented as a two-step procedure, consisting of an initial reduction to tridiagonal form, followed by computation of eigenvalues and eigenvectors by the QR algorithm. The computational complexity of the eigenvalue decomposition scales proportionally to N^3. This needs to be performed only once before the beginning of the iterative process, while at each new iteration the complexity of computing the inverse according to (5) has a more computationally amenable cost proportional to N^2.

MCMC settings

For the heritability results presented in the manuscript we used the JAGS software (Plummer, 2003) and its R interface package, rjags, to run the MCMC sampling. We used 2 chains, with an initial adaptation phase, a burn-in phase with 50 iterations and a monitoring phase with 300 iterations. We collected samples from the 300 final iterations. Convergence was determined based on Gelman and Rubin's convergence diagnostic and by visually inspecting that the chains have crossed and that the autocorrelation of subsequent samples is close to 0.

References

Plummer, M. (2003). *JAGS: A program for analysis of Bayesian graphical models using Gibbs sampling.*

Yang, J., Lee, S. H., Goddard, M. E., and Visscher, P. M. (2011). GCTA: A Tool for Genome-wide Complex Trait Analysis. *American Journal of Human Genetics*, 88(1):76–82.
